# Regulation of reactive oxygen molecules in pakchoi by histone acetylation modifications under Cd stress

**DOI:** 10.1371/journal.pone.0314043

**Published:** 2024-11-20

**Authors:** Xiaoqun Cao, Ming Zhang, Xufeng Xiao, Fengrui Yin, Yuekeng Yao, Meilan Sui, Yifan Hu, Yan Xiang, Liangdeng Wang

**Affiliations:** 1 College of Agronomy, Jiangxi Agricultural University, Nanchang, Jiangxi, P. R. China; 2 Department of Animal Science, Jiangxi Biotech Vocational College, Nanchang, Jiangxi, P. R. China; Sichuan University, CHINA

## Abstract

Reactive oxygen species (ROS) are essential modulators of epigenetic modifications under abiotic stress. However, the mutual regulation mechanism of the two under cadmium (Cd) stress is unclear. In this work, we investigated this issue using Cd-stressed pakchoi seedlings treated with six epi-modification inhibitors (5-AC, RG108, TSA, CUDC101, AT13148, and H89) as experimental materials. The experimental data showed that Cd stress caused ROS accumulation and chromatin decondensation. Addition of low concentrations of epi-modification inhibitors increased histone acetylation modification levels, and effectively attenuated cell cycle arrest and DNA damage caused by Cd-induced ROS accumulation, where histone acetylation modification levels were co-regulated by histone acetyltransferase and deacetyltransferase gene transcription. Moreover, the addition of the antioxidant Thi enhanced this mitigating effect. Also, TSA addition at high concentrations could also increase Cd-induced ROS accumulation. Based on this, we propose that the ROS molecular pathway may be related to epigenetic regulation, and chromatin modification may affect ROS accumulation by regulating gene expression, providing a new perspective for studying the regulatory mechanism of epigenetic modification under abiotic stress.

## Introduction

Heavy metal pollution, especially cadmium (Cd), has become one of the most serious environmental pollution problems in the world with the rapid development of society [1]. Cd is highly toxic, long-lived, easily absorbed and accumulated by plants, and accumulates through the food chain in humans and animals, posing a serious threat to human health [2,3]. In plants, Cd accumulation can induce ROS production by damaging mitochondria and other key organelles (e.g., chloroplasts, peroxisomes, and plasma membranes) [4]. Elevated intracellular ROS levels not only inhibit enzyme activities, causing lipid peroxidation, protein oxidation, and DNA and RNA damage [5], but also affect carbohydrate structure and disrupt signal transduction [6,7], weakening the function of plant defense systems. For example, Cd toxicity induces oxidative stress in broad bean seedlings grown after Cd treatment and recreational water stress, disorganising ROS at the genomic DNA level, ultimately leading to replication stress [8]. Interestingly, low levels of ROS increase plant tolerance, whereas high levels lead to lipid peroxidation in plant cells [9].

The eukaryotic cell cycle is a complex process divided into four successive phases: G_1_ (postmitotic interphase), S (DNA synthesis), G_2_ (postsynthesis), and M (mitosis) [10]. Any disturbance in the cell cycle, such as DNA damage, DNA replication stress, and abnormal chromosome function, can lead to a block in the process of cellular repair of defects in different phases of the cell cycle [11,12]. Recent studies have shown that reactive oxygen species (ROS) may be involved in the induction of transient G_2_/M arrest [13]. For example, in maize cells, ROS are involved in delays in the G_2_/M phase of the cell cycle caused by inhibitors of epigenetic modifications [10].

In plants, scavenging systems (e.g. antioxidants) effectively maintain ROS at optimal levels. However, when cells are stressed, the redox state is disrupted, excess oxidants are produced, and oxidative stress is induced, which affects cellular function, nuclear gene expression, and epigenetic modifications, including direct modifications and transcription factor activation [14]. For example, primary metabolites are required as substrates or cofactors for enzymes involved in histone acetylation homeostasis and are regulated at levels determined by the intracellular redox state [15]. Many studies have shown that abiotic stress affects epigenetic changes in plants [16,17]. Just as plants undergo multiple forms of chromatin modification under Cd stress, including acetylation, methylation, phosphorylation, and ubiquitination, which then affect gene expression by altering chromatin architecture and transcription factor availability [18,19]. Histone acetylation is involved in many different cytosolic processes, ranging from cell cycle progression, growth and development, DNA repair, and gene silencing, and is also co-regulated by histone acetyltransferases (HAT) and histone deacetylases (HDAC) [20]. In addition, HAT/HADC enzyme activity and acetyl coenzyme A metabolism are redox-regulated. For example, the addition of NO or GsNO in *Arabidopsis* resulted in the inactivation of the HDAC complex and increased histone acetylation [21]. In heat-induced programmed cell death, increased ROS accumulation led to overexpression of HAT in maize seedlings, thereby ensuring high acetylation [22]. In sugar beet lines, different levels of ROS expression can also directly lead to different levels of histone acetylation [23].

Excessive Cd stress limits root and stem growth in pakchoi plants, leading to the accumulation of large amounts of ROS [24] as well as significant genome-wide changes in the level of histone acetylation [25]; However, the relationship between the two is currently unknown. In recent years, many reports have shown that the use of relevant epi-modification inhibitors is an efficient way to study the regulatory mechanism between histones and ROS [26,27]. Therefore, in the current study, we used Cd-challenged pakchoi as a research material and used small molecule epi-modification inhibitors to investigate the relationship between the regulation of epi-modifications and ROS regulation, in order to identify the new pathway and possible mechanism of the regulation of epi-modifications under Cd test stress.

## Materials and methods

### Plant material

Pakchoi seeds of the ‘Short-legged Shanghai Green’ variety were soaked in warm broth and scattered in seedling trays. When the seedlings reached 4–5 leaves, the uniformly grown seedlings were selected and transplanted into moulded plastic pots containing 1 litre of Hoagland’s nutrient solution and pre-cultured for 10 days. The seedlings were then divided into three portions and treated: (1) for different Cd concentrations, such as 2, 4, 6, and 8 mg/L (designed Cd2, Cd4, Cd6, and Cd8, respectively), with nutrient solution treatment only as a control (designated Cont), (2) for the addition of six epi-modification inhibitors (5-AC, RG108, TSA, CUDC101, AT13148, and H89) based on 6 mg/L Cd treatment plus an additional antioxidant, thiourea (designated as Thi), and (3) for a Cd concentration of 6 mg/L with a high concentration of 5 μmol/L TSA (designated high TSA) (Table 1). The concentrations of the six inhibitors and Thi were 0.5 μmol/L. During the treatment period, the nutrient solution was changed every 4 days by stirring three times. Plants were harvested after 32 days. The above experiments were also randomly performed in triplicate (n = 3).

**Table 1:** Experimental design options.

Disposal No.	Experimental design
Group 1	Cont, Cd6, Cd6 + 5-AC, Cd6 + RG108, Cd6 + TSA, Cd6 + CUDC101, Cd6 + AT13148, Cd6 + H89
Group 2	Cd6 + 5-AC + Thi, Cd6 + RG108 + Thi, Cd6 + TSA + Thi, Cd6 + CUDC101 + Thi, Cd6 + AT13148 + Thi, Cd6 + H89 + Thi
Group 3	Cont, Cd6, Cd6 + high TSA

### Determination of ROS and enzymatic activities

Hydrogen peroxide assay (A064-1-1, Nanjing Jianjian Bioengineering Institute, China) and superoxide anion assay (BC1290, Solarbio, China) were determined according to the methods provided by the manufacturer. The sample (0.2 g) was weighed into a 2 mL centrifuge tube and 1.6 mL of pre-chilled phosphate solution (50 mM, pH 7.8) was added and then centrifuged at 4°C and 12000 rpm for 20 min. The supernatant was used as a crude extract of the enzyme and stored at 4°C for activity assay. An ultraviolet (UV) spectrophotometer (Huang visible spectrophotometer, UV-2600, Shimadzu, Kyoto, Japan) was used to determine POD and CAT. In addition, a multifunctional enzyme marker (SpectraMax® M2, Molecular Devices, USA) and a plant malondialdehyde ELISA kit (JN24318, Shanghai Jining Biological Co., Ltd., China) were used to measure the SOD activity and MDA, respectively.

### Determination of Cd by ICP-MS

Weigh 0.2 g into the microwave digestion vessel, add 5 to 10 mL of nitric acid, cover the vessel, and leave for at least 1 hour, tighten the lid of the vessel and digest according to the standard operating procedure for microwave digestion apparatus (EVISA, Upper Cohen, Germany). After digestion, allow the pot to cool to room temperature, slowly open the inner pot, then open the lid to release the gas inside, wash the lid twice with a small amount of distilled water, and allow the wash water to flow back. Place the pot on a temperature-controlled hotplate, heat to 120°C and leave for 30 minutes. Transfer all the removed solution to a 50 mL volumetric flask and then add 3% dilute nitric acid to make up the volume. Shake well to use as the test solution and perform a blind test at the same time. The ICAP Q ICP-MS parameters (Thermo Scientific, Waltham, MA, USA) were configured as follows: pump speed (40 rpm), radio frequency power (1550 W), S/C temperature (2.7°C), sample depth (5 mm), cooled flow rate (14 L/min), and auxiliary flow rate (0.8 L/min), auxiliary flow rate (0.5 L/min), and nebuliser flow rate (0.9 L/min).

### Western blot

Proteins were extracted according to the method of Li *et al*. [28]. First, 0.5 g of leaves were quickly crushed, and then extraction buffer [100 mM Tris-Cl pH 7.4, 50 mM NaCl, 5 mM EDTA, and 1 mM phenylmethylsulfonyl fluoride (PMSF)] was added. The supernatants were then collected and analysed by Western blotting according to Niogret *et al*. [29] analysed. Histone H3 was used as an in-band reference throughout the protein immunoblotting assay; In addition, anti-H3K9ac antibodies (ab32129) and anti-H4K5ac antibodies (ab51997) were purchased from Abcam (Cambridge, UK), while anti-H3ac antibodies (05–724) were purchased from Millipore (Massachusetts, USA). In addition, an AP-conjugated goat anti-rabbit IgG secondary antibody (A0208) was purchased from Beyotime (Beijing, China). Three independent assays were performed for H3K9ac, H4K5ac, and H3ac, and the analysis included the control, Cd groups, and later inhibitor treated groups.

### Immunostaining

Nuclear isolation and staining were performed as previously described. Briefly, nuclei were flattened on slides and incubated with 3% BSA, primary and secondary antibodies, respectively, followed by DAPI staining, Vectashield mounting, and photographic observation using an Olympus BX-60 fluorescence microscope and DAPI fluorescein filter blocks. Images captured with a Sensys 1401E CCD monochrome camera (Olympus, Tokyo, Japan) were pseudocoloured using METAMORPH 4.6.3 software (Universal Imaging Corp., Downingtown, PA, USA). The settings were consistent for each channel. Over 500 nuclei were retained in each processing group.

### TUNEL and γ-H2AX immunostaining assays

To complete the apoptosis test, the TUNEL staining kit and the γ-H2AX immunoassay kit from Roche (USA) were used. The paraffin sections were prepared from the root tip of pakchoi, and the images were collected by dewaxing, proteinase K repair, membrane rupture, equilibration at room temperature, addition of reaction solution, DAPI staining of cell nuclei, shaking, and washing in PBS solution on a destaining shaker, sealing the sections with an autofluorescence quencher, and finally observing of the sections under a fluorescence microscope.

### Cell cycle assay

The test device was a CyFlow Space flow cytometer (Sysmex Partec, Germany). The analysis kit was the CyStain UV Precise P Ploidy Analysis Kit (SysmexPartec, Germany). Take 0.2 g of fresh pakchoi sample and place it in a Petri dish. Add 500 μL of nuclear lysis solution around the sample and crush it with a sharp blade to completely extract the intact nucleus for 60 s. Filter the liquid in the Petri dish into a sample tube using a 50 μm filter. Add 2000 μL of fluorescent staining solution to the sample tube and stain for 2 minutes in the dark. Analyse the image. The fluorescence intensity is proportional to the DNA content, which can be derived from the number and proportion of cells in different division phases.

### Real-time quantitative PCR

The Trizol kit extracted total RNA from the samples, which was reverse transcribed into cDNA using the TaKaRa Reverse Transcription Kit (RR420A), and the amount of cDNA template was adjusted so that the amount of cDNA was consistent for each treatment. According to the target gene sequences, quantitative PCR primers were designed separately (Table 2), and qPCR SYBR Green Master Mix Fluorescence Quantification Kit (RR047A) was used to perform qRT-PCR in a quantitative PCR amplifier to identify the target gene, an internal gene detection reference gene Ct values of each sample were repeated three times.

**Table 2:** qRT-PCR primers information.

Gene name	Primer sequence (5’-3’)
*GCN5*-F *GCN5*-R	CGGAACACTAAGATTAAGACG CATACAAACTTGAGCCGATTA
*HAT-B*-F *HAT-B*-R	CAGATCTTGGTCCTGCCTTC GGAACTTTTCGGATGGCTCT
*HDAC101*-F *HDAC101*-R	CCAATGGGAAAGTACATCGAAAGGTGG GAACCACACATCTTTGAGTGACTCCGG
*β-ACTIN*-F	GGAGCTGAGAGATTCCGTTG
*β-ACTIN*-R	GAACCACCACTGAGGACGAT

### Statistical analysis

At least three replicates were performed for each experiment. SPSS 22.0 software was used for statistical analysis of all data, which were presented as mean ± standard deviation (SD) for each treatment. The least significant difference (LSD) post hoc test was performed at 5% significance levels to identify differences between individual treatments (p < 0.05).

## Results

### Cd-induced ROS accumulation and chromatin decondensation

**Fig 1:**
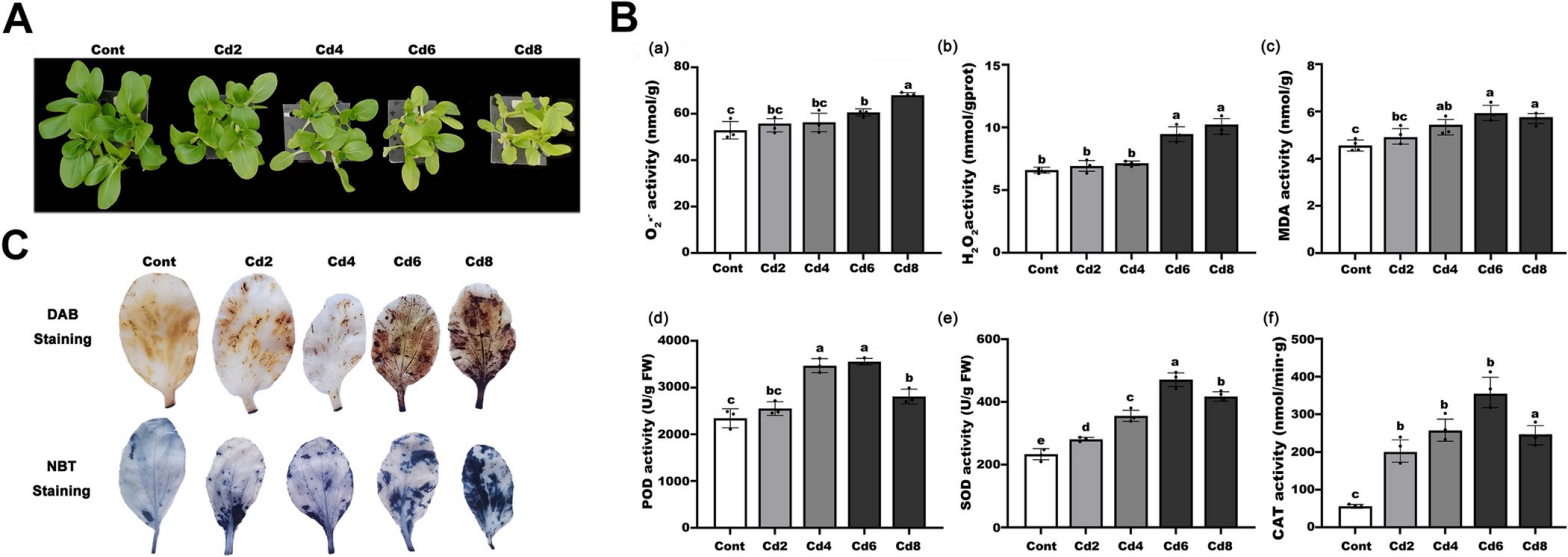
Growth of pakchoi seedlings exposed to Cd concentrations for 32 days. (A) The phenotypic identification of pakchoi seedlings. (B) ROS accumulation and relevant physiological indicators, including O_2_·^**-**^ levels (a), H_2_O_2_ levels (b), MDA content (c), and enzyme activities of POD (d), SOD (e), CAT (f) into leaves. (C) DAB and NBT staining in the large leaves. Data are presented as mean ± SD (n = 3). Statistically significant differences indicated with different letters at p < 0.05 level.

Cd could induce ROS accumulation, including H_2_O_2_ and O_2_·^-^, and both were increased in a density-dependent manner (Fig 1A and 1B), a result similarly demonstrated by DAB and NBT polymerisation (Fig 1C). Incubation with Cd^2+^ resulted in the typical brown and blue precipitates of the cytoplasm and nuclei, particularly at Cd levels of 6 mg/L and 8 mg/L. In addition, antioxidant enzyme activities were markedly increased (p<0.05), as was the MDA content, all of which correlated positively with Cd dose.

**Fig 2:**
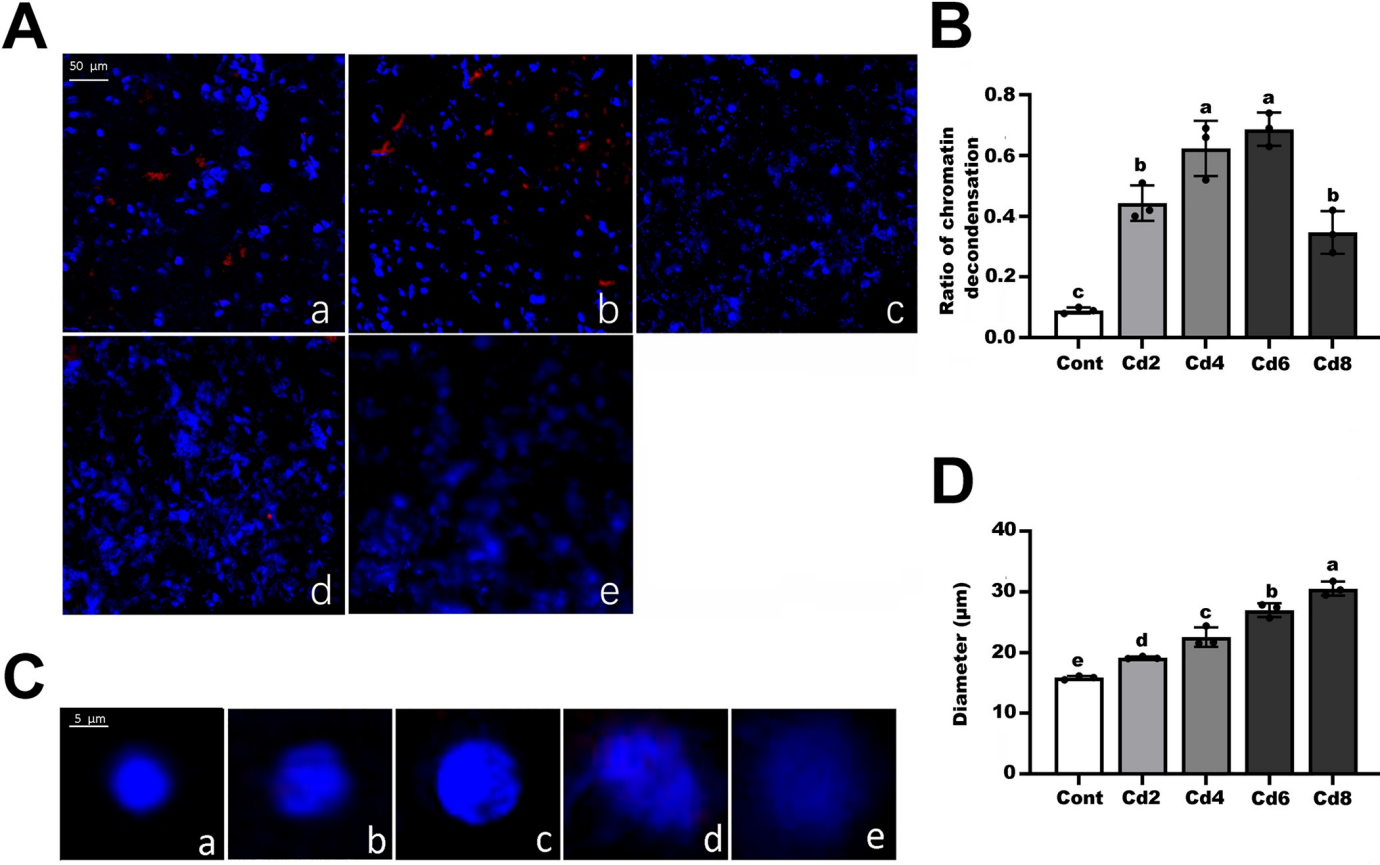
Observation of chromatin decondensation levels induced by different Cd concentrations in pakchoi. (A) Chromatin decondensation of representative interphase nuclei from different Cd concentration treatments. (a)–(e) represent Cont, Cd2, Cd4, Cd6, and Cd8 groups, respectively. (B) Quantitative analysis of immunostaining results in (A) using Metamorph software. (C) Nucleolus size increased during Cd treatment. (a)–(e) equally represent Cont, Cd2, Cd4, Cd6, and Cd8 groups. (D) Statistical analysis of nucleolus size. Nuclei were stained with DAPI (blue). More than 400 nuclei were analysed for each antibody. Data are presented as mean ± SD (n = 3). Statistically significant differences indicated with different letters at p < 0.05 level.

Additionally, fluorescence immunostaining indicated that the nuclei of leaf cells gradually disintegrated from intact rounded to irregularly dispersed as Cd concentration increased, which was significantly larger (Fig 2A–2D). The relative amount of cellular chromatin decondensation increased from less than 8% in the control group to 66% in the Cd6 group, but the Cd8 group showed a decreasing trend, which we believe may be related to the severe destruction of the cellular structure by high Cd concentration.

### Addition of epi-modification inhibitors reduced Cd-induced ROS accumulation

**Fig 3:**
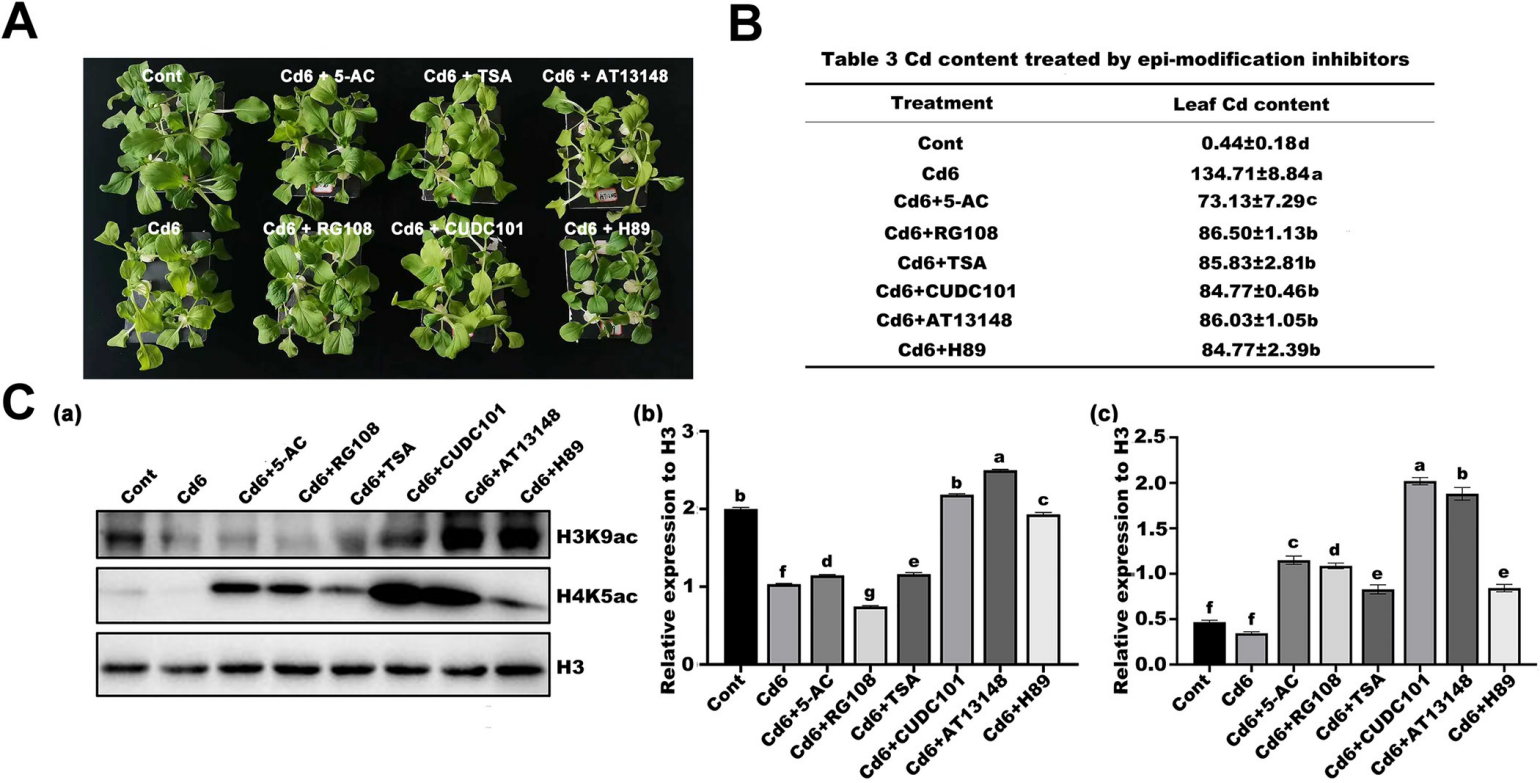
Growth of pakchoi seedlings exposed to Cd concentrations of 6 mg/L plus six low concentrations of epi-modification inhibitors. (A) The phenotypic identification of pakchoi seedlings exposed to Cd concentrations of 6 mg/L plus six low concentrations of epi-modification inhibitors 5-AC, RG108, TSA, CUDC101, AT13148, and H89 for 32 days. (B) Cd accumulation. (C) Antibodies specific for H3, H3K9ac, and H4K5ac were used to analyse histone extracts from pakchoi seedlings. (b)–(c) Quantitative analysis of H3K9ac and H4K5ac in (a) Image J. Data are presented as mean ± SD (n = 3). Statistically significant differences indicated with different letters at p < 0.05 level.

**Fig 4:**
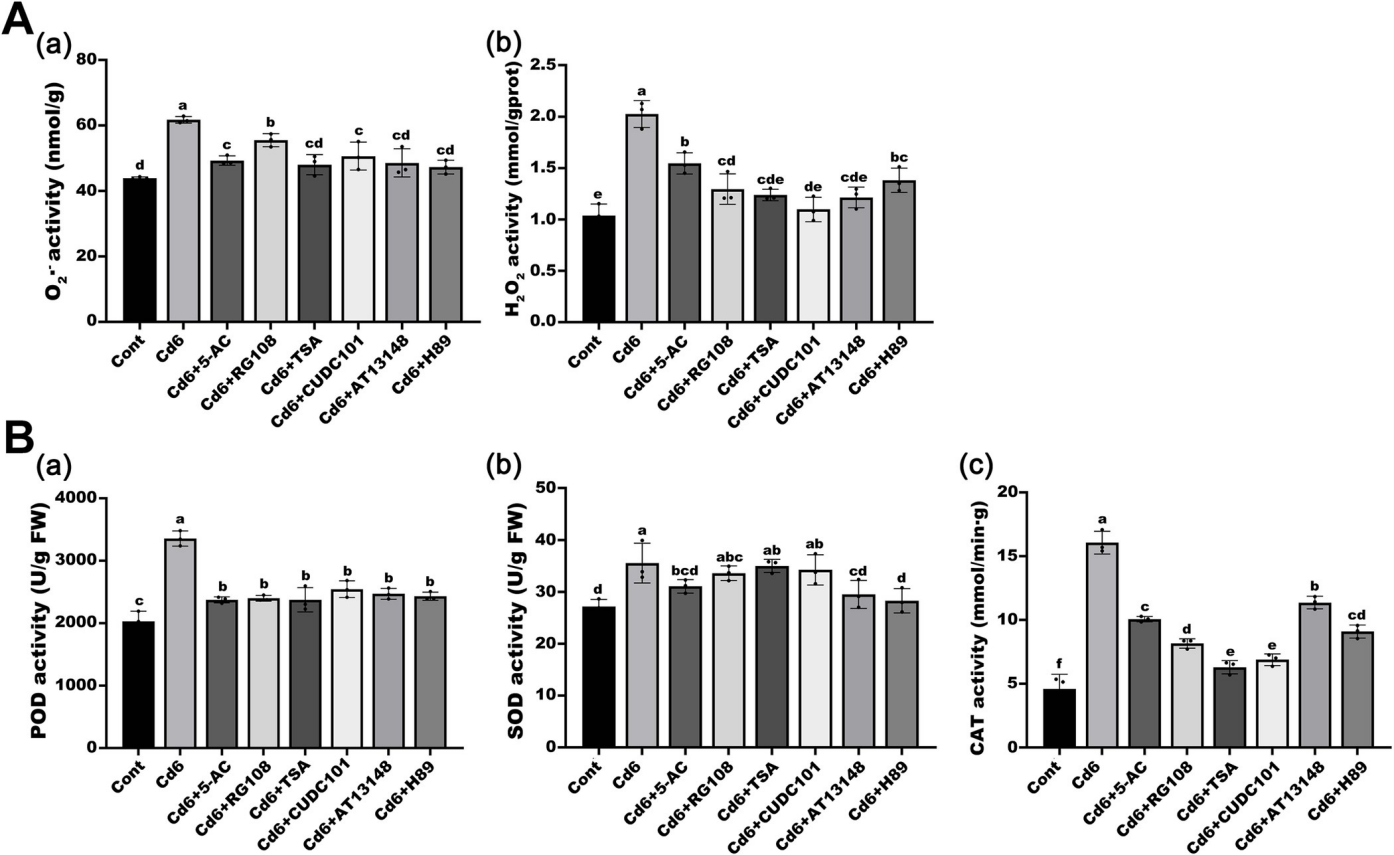
Effect of low concentrations of epi-modification inhibitors on the response mechanism of the antioxidant system under Cd stress. (A) Concentration of O_2_·^-^ (a) and H_2_O_2_ (b). (B) Enzyme activities of POD (a), SOD (b), and CAT (c) in leaves. Data are presented as mean ± SD (n = 3). Statistically significant differences indicated with different letters at p < 0.05 level.

To investigate how epigenetic modifications affect the growth and development of pakchoi seedlings under Cd-stressed conditions, we sequentially added six epi-modification inhibitors (5-AC, RG108, TSA, CUDC101, AT13148, and H89) to the Cd6 group to treat seedlings for 32 d (Fig 3A). Of these, H89 and AT13148 are histone phosphorylation inhibitors, 5-AC and RG108 are DNA methylation inhibitors, and TSA and CUDC101 are histone deacetylation inhibitors. We first examined the Cd content in pakchoi leaves in each treatment group and found that all six inhibitor additions significantly reduced Cd accumulation compared to the Cd6 group (Fig 3B). Subsequently, Western blot assay showed that under Cd stress, all inhibitors led to an obvious increase in the acetylation levels of histone H4K5 and H3K9 sites, except for H3K9ac, which showed no obvious changes in the RG108 treatment; among them, the levels of H4K5ac even increased more than 2-fold in all the inhibitor treatments compared to those in the Cd6 group (Fig 3C), suggesting that low concentrations of epi-modification inhibitors can induce specific histone acetylation changes in pakchoi under Cd stress. To better understand the role of ROS role in this process, we also examined the molecular levels of ROS in each treatment group. The results showed that, although the treatment with these inhibitors still led to an increase in intracellular oxygen pressure compared to the control, the use of these inhibitors not only reduced the accumulation of ROS (Fig 4A) but also showed a similar decreasing trend in the activities of related enzymes (Fig 4B), suggesting that there may be a link between the accumulation of ROS and the changes in histone acetylation.

### Addition of epi-modification inhibitors alleviated Cd-induced cell cycle arrest

**Fig 5:**
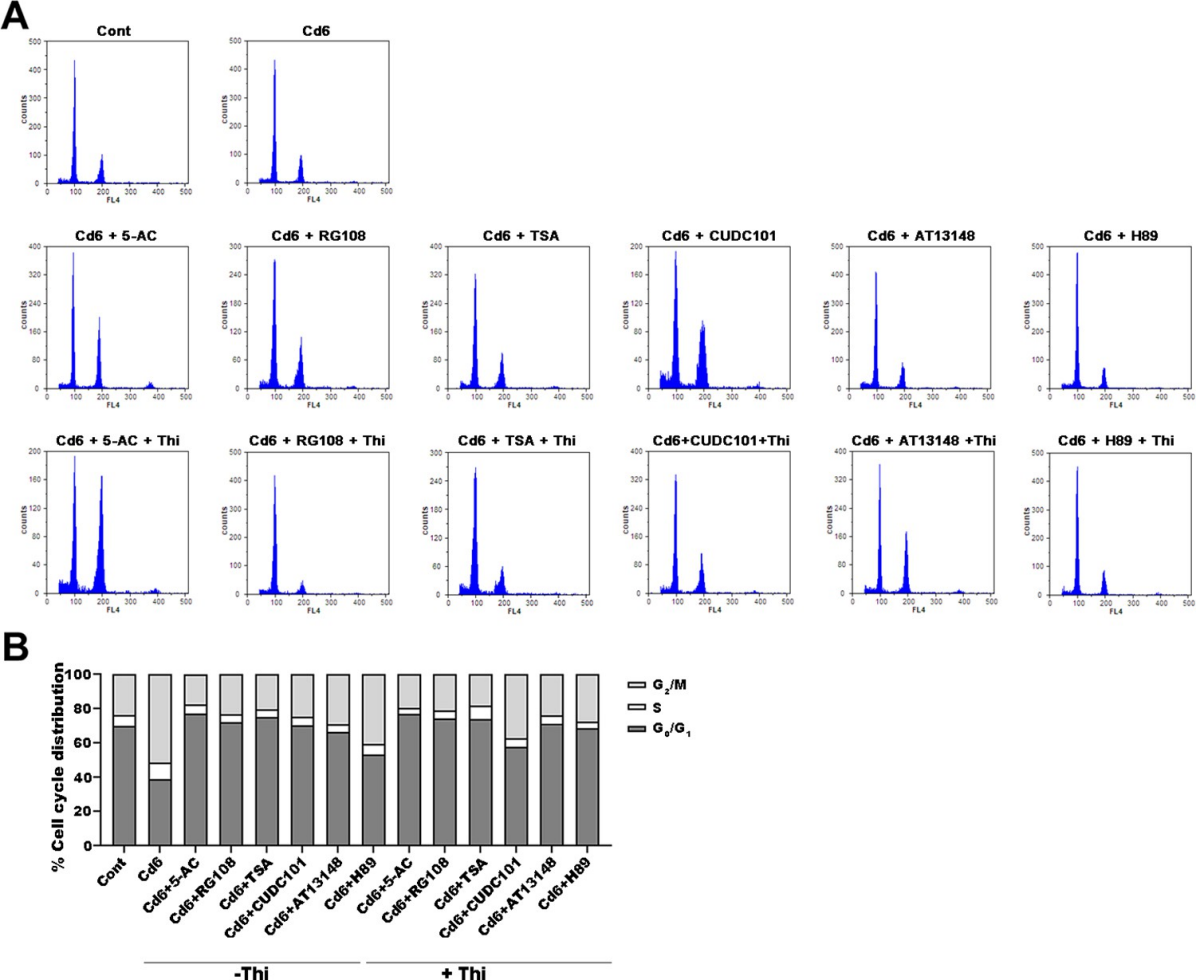
Effect of low concentrations of epi-modification inhibitors on Cd stress-induced cell cycle arrest. (A) Flow cytometry results for ploidy levels treated with six low concentrations of epi-modification inhibitors 5-AC, RG108, TSA, CUDC101, AT13148, and H89 plus additional Thi for 32 days under Cd stress. (B) Percentage of cells in G_1_, S, and G_2_ phase detected by flow cytometry.

Cell cycle arrest usually occurs in the G_1_/S and G_2_/M phases of mitosis. Here, we analysed the effects of six inhibitor treatments on Cd-induced cell cycle arrest using flow cytometry and FLOWJO software. The findings showed that the percent of nuclei in the G_2_ phase was increased in the Cd6 group compared to the control, suggesting that Cd induces the accumulation of the G_2_ phase in pakchoi leaf cells (Fig 5A and 5B). Interestingly, compared with the Cd6 group, all the six epi-modification inhibitors addition differently reduced the percentage of cells in the G_2_ phase, suggesting that the addition of these inhibitors significantly alleviated the Cd stress-mediated cell cycle blockade in pakchoi leaf, which we consider may be related to the reduced ROS accumulation detected earlier (Fig 5B). To verify whether ROS were involved in these cell cycle blocks, we added antioxidant, Thi, to the plants in each treatment group to reduce the concentration of H_2_O_2_ in the leaf cells, and, unsurprisingly, we finally found that the addition of Thi further alleviated the blockade on the G_2_ phase cells (Fig 5B), suggesting that low concentrations of the epi-modification inhibitor can affect ROS accumulation and thus alleviate Cd-induced cell cycle block.

### Accumulation of ROS molecules directly affected genomic DNA damage

**Fig 6:**
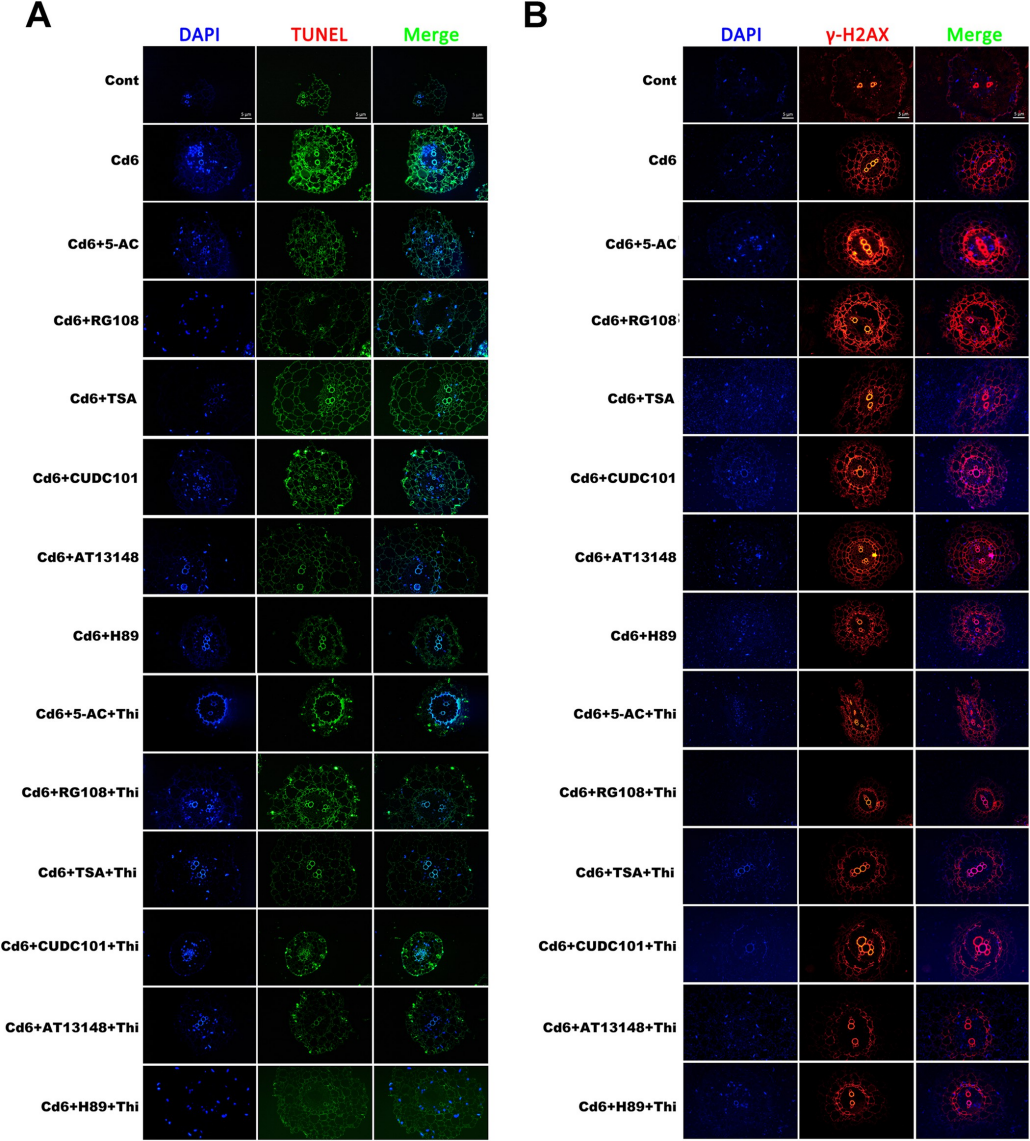
Low concentrations of epi-modification inhibitors attenuated Cd-induced DNA damage and Thi acted synergistically. (A) Detection by TUNEL assay. (B) Detection of the γ-H2AX signal. Cell nuclei were stained with DAPI (blue).

Previously, we showed that chromatin in pakchoi leaf cells under Cd stress underwent significant decondensation and accumulation of ROS molecules and that the addition of low concentrations of epi-modification inhibitors effectively reduced Cd-induced ROS accumulation. As a result, we detected DNA damage in leaf cells of pakchoi seedlings from the Cd group after treatment with epi-modification inhibitors using the TUNEL assay and γ-H2AX immunostaining. Among them, Cd6 group plants showed typical positive results in TUNEL (Fig 6A) and γ-H2AX immunostaining assays (Fig 6B) compared to the control, indicating that Cd leads to genomic DNA breaks and DNA repair does not occur. However, plants to which the inhibitor was added showed a significant increase in the number of normal nuclei and an increase in the γ-H2AX signal, suggesting that Cd-induced DNA damage was reduced at this time and repair was initiated. In addition, the proportion of normal nuclei increased more significantly in all Cd-treated groups after the addition of the antioxidant Thi, suggesting that Cd-induced DNA damage can be effectively mitigated by changes in histone acetylation, which may be related to ROS accumulation.

### Low concentrations of epi-modification inhibitors regulated the expression of histone acetylation-related genes under Cd stress

**Fig 7:**
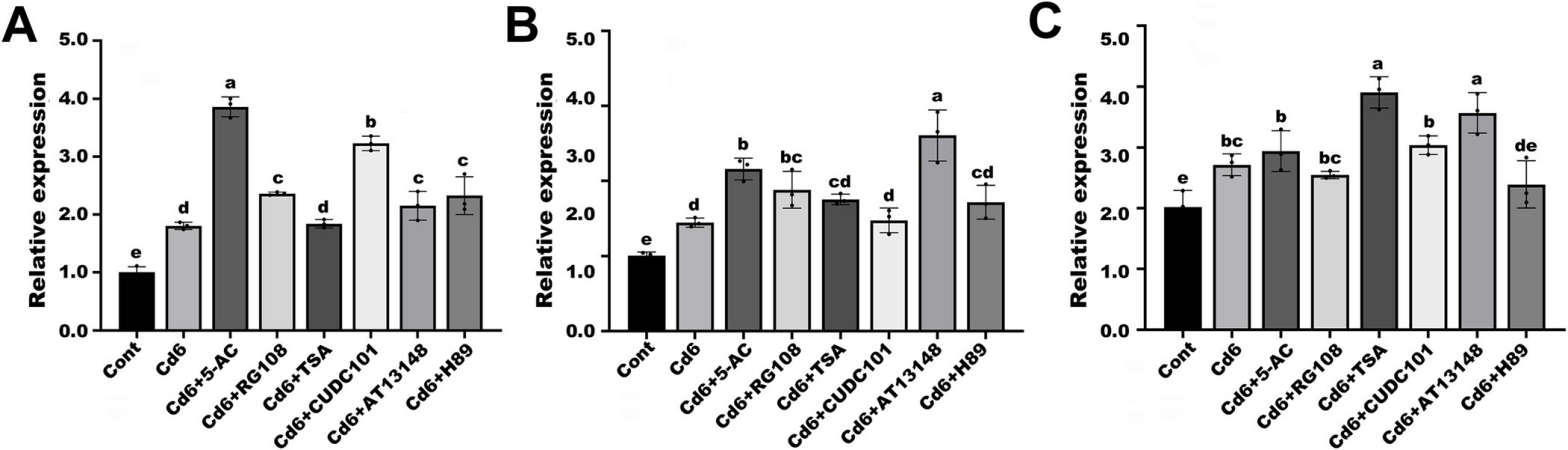
Effect of low concentrations of epi-modification inhibitors on the expression levels of genes related to histone acetylation. (A) *GCN5*. (B) *HADC101*. (C) *HAT-B*. The expression levels of all genes in the root were measured by qPCR of mRNA prepared from the control group, the Cd6 group, and different treatment groups with epi-modification inhibitors. *β-ACTIN* was used as an internal control. Data are presented as mean ± SD (n = 3). Statistically significant differences indicated with different letters at p < 0.05 level.

To analyze the transcription of genes encoding histone acetylation upon Cd co-treatment with low concentrations of epi-modification inhibitors, we examined the transcription of two representative histone acetyltransferase genes (*GCN5* and *HAT-B*) and a histone deacetylase gene (*HDAC101*). The results showed that transcripts of the *HDAC101* gene were significantly increased after the addition of low concentrations of epi-modification inhibitors compared to the Cd6 and untreated groups, with an approximately 2-fold increase in expression after treatment with the inhibitor 5-AC (Fig 7A). In addition, the transcripts of the *GCN5* and *HAT-B* genes were also significantly up-regulated, with the transcripts of the *GCN5* gene reaching a maximum after treatment with the inhibitor AT1318 and the transcripts of the *HAT-B* gene reaching a maximum after treatment with TSA, suggesting that the changes in histone acetylation modulate through gene transcription and thus alleviate Cd-induced DNA damage (Fig 7B–7C).

### Histone acetylation regulates Cd stress-induced ROS accumulation

**Fig 8:**
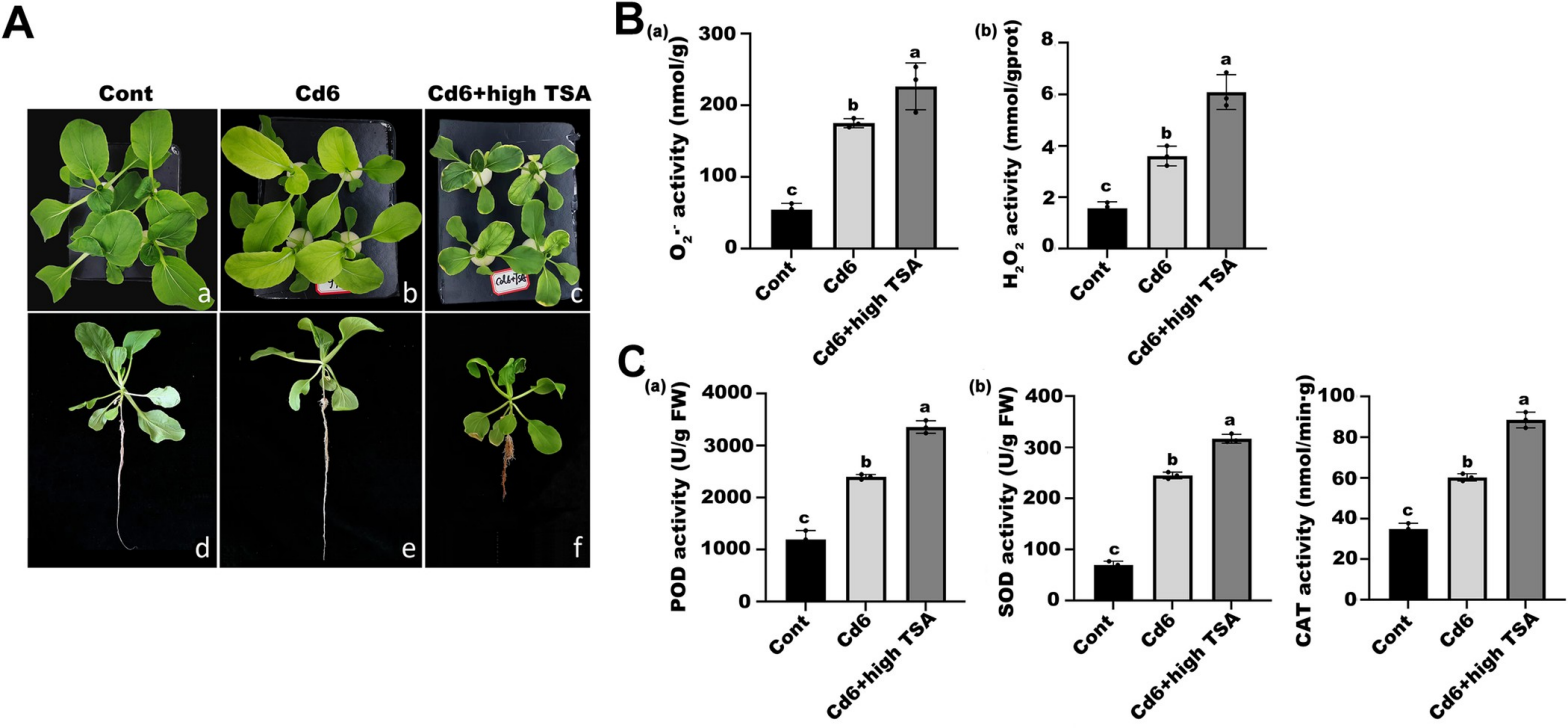
Variations of ROS-related molecules and enzyme activities under Cd concentration of 6 mg/L plus high TSA concentration. (A) Plant phenotypes of aboveground (a)–(c) and individual seedlings (d)–(f) of pakchoi in the Cont, Cd6, and Cd6 + high TSA groups, respectively. (B) Concentration of O_2_·^-^ (a) and H_2_O_2_ (b). (C) Enzyme activities of POD (a), SOD (b), and CAT (c) in leaves. Data are presented as mean ± SD (n = 3). Statistically significant differences indicated with different letters at p < 0.05 level.

Previous studies have shown that low concentrations of TSA treatment resulted in elevated H3K9ac and H4K5ac, which is generally consistent with the significant increase in histone acetylation at the genomic level under co-treatment of other epi-modification inhibitors with Cd. Therefore, we co-treated pakchoi seedlings with high concentrations of TSA and Cd6 to simulate a state of high histone acetylation and further investigate its relationship with ROS accumulation (Fig 8A and 8B). We found that the levels of H_2_O_2_ and O_2_·^**-**^ in the leaves of pakchoi seedlings co-treated with high concentrations of TSA and Cd were considerably higher than those in the Cd6 group and the untreated group (Fig 8B), and the levels of the ROS-related enzymes POD, SOD, and CAT were also significantly increased (Fig 8B). Based on this, we suggest that this histone hyperacetylation state might have exacerbated ROS accumulation in leaf cells under Cd stress.

## Discussion

Although the understanding of the role and biological significance of reactive oxygen metabolites is progressing, and the main understanding has shifted from destructive effects to signal transmissions in general [30], the prevailing view remains unchanged and indicates a dual dependence of ROS on concentration. ROS are thought to be engaged in signalling pathways even at low doses, in response, for example, to pathogen attack; ROS overproduction and breakdown of redox homeostasis causes reactions with all types of organic molecules [31]. Of all the heavy metals, Cd has been identified as a very hazardous stressor, disrupting intracellular redox homeostasis. Previous findings have demonstrated that Cd modulates membrane-located NADPH oxidase, which in turn contributes to increased ROS production, resulting in the formation of oxidative stress [32,33]. In our study, we again demonstrated that pakchoi seedlings are susceptible to Cd stress, and both the accumulation of ROS molecule and related enzyme activities showed a dose-dependent relationship with Cd concentration, especially reaching a maximum value at 6 mg/L, which again proved that Cd stress affected the redox system of pakchoi cells and caused oxidative stress in them. Our histochemical analysis using DAB and NBT staining also well supported this conclusion. Similar results were previously obtained in studies on *V*. *radiata* [34], *B*. *napus* [35], *B*. *juncea* [36], *A*. *thaliana* [33], and *Cucumis sativus* [37], among other plant models. Later, we found that the chromatin structure gradually became looser with increasing Cd concentration, the nuclei of root cells were enlarged and the chromatin was diffuse starting from the 6 mg/L Cd group, indicating that Cd stress can depolymerise chromatin in root cells, and this depolymerisation was dose-dependent with the Cd concentration, similar to the response of maize under heat stress in our results [38]. Typically, chromatin remodelling is strongly linked to epigenetic modifications of histones (e.g. demethylation/methylation and acetylation/deacetylation) that can change chromatin structure and lead to activation or repression of transcription. For example, in *Arabidopsis*, the heterotrimeric CAF-1 hapten complex targets acetylated histone H3/H4 to nascent DNA strands for nucleosome recombination [39]. Based on this, we hypothesise that Cd-induced oxidative stress leads to ROS accumulation with chromatin decondensation, both of which may be associated with changes in epigenetic modifications.

We therefore used two phosphorylation inhibitors, two DNA methylation inhibitors, and two histone acetylation inhibitors in combination with Cd treatment in pakchoi seedlings. Our results showed that both H_2_O_2_ and O_2_·^**-**^ levels were reduced in pakchoi root cells after treatment with six epi-modification inhibitors, as well as the corresponding antioxidant enzyme activity, which might be related to the reduction of Cd accumulation. However, the acetylation levels of both H3K9 and H4K5 sites showed a significant increase, suggesting a possible correlation between ROS accumulation and changes in histone acetylation modification. In maize, heat stress resulted in increased acetylation, decreased methylation, increased ROS accumulation, nuclear enlargement, and chromatin decondensation [22]. Similarly, in *Arabidopsis*, adding SA and flg22 to cell suspension cultures resulted in an increase in ROS and the oxidation of two deacetylases, HDA9 and HDA19, leading to ameliorated stress-responsive gene acetylation [40]. All of these results suggest a concomitant relationship between ROS accumulation and changes in epigenetic modifications, but it remains unclear as to which is upstream.

In *Arabidopsis*, glutathione (GSH) plays a crucial role in *Arabidopsis* cell cycle progression [41]. At the same time, ascorbic acid-deficient, and vitamin C-deficient (vtc) *Arabidopsis* mutants exhibit mild oxidative stress in the nucleus, and cell cycle progression is impaired [42]. Our results showed that low concentrations of epigenetic inhibitor treatments were also effective in alleviating Cd stress-induced G_2_ phase cell cycle arrest, while additional antioxidant Thi enhanced the inhibitory effect, suggesting that histone acetylation modifications and antioxidant effects synergistically regulate Cd-induced cell cycle arrest. In an attempt to examine the relation between histone acetylation and ROS accumulation, we used the histone deacetylase inhibitor TSA to simulate a state of high histone acetylation in leaf cells of pakchoi seedlings under Cd stress, which caused the accumulation of intracellular ROS, indicating that histone acetylation can regulate the accumulation of intracellular ROS under Cd stress, which is consistent with the results reported for maize [22]. ROS accumulation induces DNA damage and DNA repair [43], whereas the DNA damage response (DDR) leads to cell cycle arrest, DNA repair, or apoptosis [44]. In the present study, we found that DNA damage was attenuated by the addition of epi-modification inhibitors, and the alleviation of this damage symptom was enhanced by the addition of the antioxidant Thi, suggesting that ROS molecules are the direct cause of genomic DNA breaks and that histone acetylation may play a role in regulating ROS accumulation.

Numerous studies have demonstrated that histone acetylation is relevant to transcriptional activation and histone deacetylation is relevant to transcriptional silencing. In many plant species, the transcription of stress-responsive genes is linked to dynamic changes in histone acetylation, like *Arabidopsis* [27,45,46], rice (*Oryza sativa*) [47,48], and maize (*Zea mays*) [49]. Normally, plant HATs and HDACs respond to environmental stresses and participate in plant development by regulating gene expression. Moreover, they are involved in the transcriptional regulation of genes during a variety of developmental processes in cooperation with different chromatin remodelling factors and transcription factors [50]. In our study, the acetylation level of histone H4K5 sites was significantly more than that of the Cd6 and untreated groups after treatment with a low concentration of the table modification inhibitor, while the acetylation level of histone H3K9 sites was higher than that of the Cd6 group but lower than that of the untreated group in general. Also, our transcripts of histone acetylation-related genes *HDAC101*, *GCN5*, and *HAT-B* were also significantly increased after treatment with low concentrations of epi-modification inhibitors, which is the same as in chickpea shoots, i.e., higher enrichment of H3K9ac was observed in the promoter region of CaHDZ12 after 24 h of mannitol treatment, suggesting that mannitol has been demonstrated to be a drought-tolerant by decreasing ROS accumulation [51]. In alfalfa, relatively high enrichment of H3K4me3 and H3K9ac was observed at the MsMYB4 promoter from 6 h after salt stress, while H3K14ac and H3K36me2 levels were maintained [52]. These results all suggest that changes in epigenetic modifications can regulate the accumulation of ROS molecules. In addition, the large intracellular accumulation of ROS molecules and the elevation of related enzyme activities found in the histone hyperacetylation mimicry assay are also a strong indication that histone acetylation modifications may regulate the ROS molecule pathway through chromatin structure and gene expression.

## Conclusion

Accumulation of ROS molecules and chromatin decondensation in pakchoi cells under Cd stress caused cell cycle arrest and led to DNA breaks. The addition of a low concentration of epi-modification inhibitor treatment elevated histone acetylation modification levels and reduced Cd-induced ROS accumulation, thereby alleviating the cell cycle arrest and DNA damage. Furthermore, addition of the antioxidant Thi further attenuated the effect. Therefore, we speculate that ROS molecules correlate with the regulation of histone acetylation modification, which is transcriptionally regulated by histone acetyltransferases and histone deacetylases.

## Supporting information

S1 File (DOCX)

S2 File (ZIP)
