# Supplementary figures and images for: Regulation of reactive oxygen molecules in pakchoi by histone acetylation modifications under Cd stress
Source: PLoS One. 2024 Nov 20;19(11):e0314043. doi: 10.1371/journal.pone.0314043 (PMC11578466; doi:10.1371/journal.pone.0314043)

- 1: Cd0
- 2: Cd6
- 3: Cd6+5AC
- 4: Cd6+RG108、
- 5: Cd6+TSA
- 6: Cd6+CUDC101
- 7: Cd6+AT13148
- 8: Cd6+H89
- 磷酸化 7:6+AT13148、8:6+H89


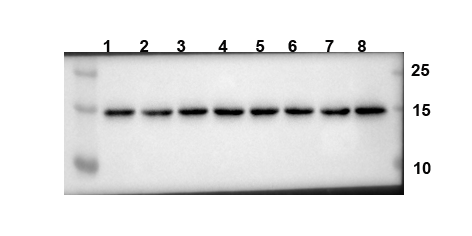

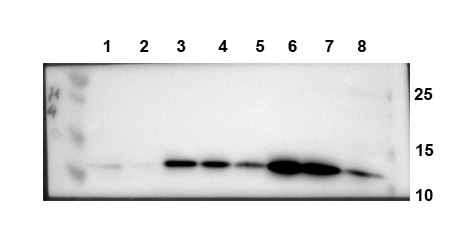

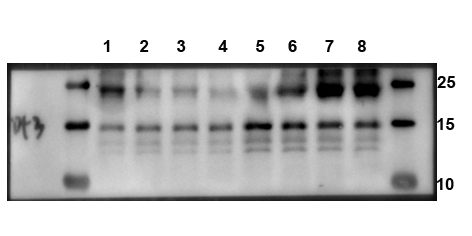

Supplement: S1 File — (DOCX) [file pone.0314043.s001.docx]
